# Molecular Gatekeepers of Aqueous Outflow: From Mechanotransduction to Gene Therapy in Trabecular Meshwork Health and Disease

**DOI:** 10.7759/cureus.91633

**Published:** 2025-09-04

**Authors:** Priti Singh, Samendra Karkhur, Vidhya Verma, Saroj Gupta, Arushi Beri

**Affiliations:** 1 Ophthalmology, All India Institute of Medical Sciences, Bhopal, IND

**Keywords:** extracellular matrix, gene therapy, glaucoma, intraocular pressure, mechanotransduction, minimally invasive glaucoma surgery, oxidative stress, rock inhibitors, stem cell therapy, trabecular meshwork

## Abstract

The trabecular meshwork (TM) plays a pivotal role in regulating aqueous humor outflow and maintaining stable intraocular pressure (IOP). When its function becomes impaired, it is a key factor in the development of primary open-angle glaucoma and other outflow disorders. Current therapies, such as topical medications, laser procedures, and surgery, can effectively lower IOP, but they rarely target the underlying cellular and molecular damage within the TM. Emerging approaches are shifting focus from bypassing to restoring TM function. These include Rho-associated protein kinase (ROCK) inhibitors, which help relax the TM; gene-based strategies such as clustered regularly interspaced short palindromic repeats (CRISPR)-Cas9 and small interfering ribonucleic acid (siRNA), which aim to correct or silence disease-driving genes; and stem or progenitor cell transplantation to replenish damaged tissue. Combining these modalities with minimally invasive glaucoma surgeries (MIGS) may further improve long-term outcomes by reducing scarring and supporting TM health. As these innovations rapidly evolve, there is a strong need for a comprehensive review to bring together existing evidence, assess their translational potential, and provide direction for future clinical applications. Collectively, such advances signal a paradigm shift in glaucoma management, from simply reducing pressure to actively restoring the TM’s intrinsic ability to self-regulate, ultimately offering hope for the sustained preservation of vision.

## Introduction and background

Glaucoma remains one of the leading causes of irreversible blindness worldwide. According to recent estimates, approximately 80 million people were living with glaucoma globally in 2024, and this number is projected to rise to over 111 million by 2040 [1]. These updated figures highlight the growing public health burden of glaucoma and underscore the need for continued research into trabecular meshwork (TM)-targeted interventions and other innovative therapeutic approaches. The TM, located at the junction of the cornea and iris, serves as the primary regulator of aqueous humor drainage and is therefore a key determinant of intraocular pressure (IOP). Aqueous humor is produced by the ciliary body, flows through the posterior chamber into the anterior chamber, and exits primarily via the TM-Schlemm’s canal pathway, with a smaller fraction draining through the uveoscleral route. This dynamic circulation maintains optical clarity and provides metabolic support to avascular structures such as the cornea and lens. Structurally, the TM comprises three interconnected regions - the uveal, corneoscleral, and juxtacanalicular (JCT) tissues - which function in concert to maintain fluid balance within the eye. When the TM loses its structural or functional integrity, aqueous outflow is impaired, leading to sustained elevations in IOP, a hallmark of primary open-angle glaucoma (POAG). Given that POAG remains the leading cause of irreversible blindness worldwide, the study of TM biology has become a central focus in ophthalmic research [2]. This review underscores the importance of targeting TM dysfunction directly, thereby shifting the paradigm of glaucoma therapy from merely lowering IOP to actively restoring TM health. Nonetheless, significant challenges remain in translating these molecular insights into clinical practice, including uncertainties regarding long-term safety and efficacy of ROCK inhibitors, delivery and off-target concerns with gene-editing technologies, and barriers to the survival and integration of transplanted stem/progenitor cells. Addressing these limitations through rigorous preclinical studies and clinical trials will be essential for the successful adoption of TM-restorative strategies.

Traditional glaucoma treatments, both drug-based and surgical, are mainly aimed at lowering IOP. Although effective in the short term, they do not individually fix the molecular and cellular disruptions within the TM that underlie disease progression. As an illustration, drugs like prostaglandin analogs and β-blockers augment aqueous humor outflow or diminish production, but they never reverse trabecular damage. Equivalently, surgical interventions, such as trabeculectomy and MIGS, circumvent or augment fluid outflow but cannot reconstitute normal TM physiology. This therapeutic deficit underscores the necessity for targeted therapy aimed at addressing the intrinsic cellular dysfunction of the tissue itself [3].

Recent findings have transformed the view of the TM from a passive sieve to an active, mechanosensitive tissue that can react to pressure fluctuations and mechanical stress. Mechanotransduction signals mediated by integrins, focal adhesions, and channels like TRPV4 and Piezo-type mechanosensitive ion channel component 1 (Piezo1) control cytoskeletal structure, contractility, and extracellular matrix (ECM) remodeling. Abnormalities in these signaling pathways lead to stiffening of the TM and increased outflow resistance. Furthermore, oxidative stress and mitochondrial impairment have also been found to advance TM cell aging, where senescent cells produce proinflammatory and profibrotic mediators that further impair outflow. Massive-throughput molecular analyses, such as transcriptomic and proteomic profiling, have extended the understanding of the TM's regulatory networks by the discovery of disease-associated proteins, genes, and pathways. Beyond providing new disease biomarkers for diagnosis, they open up new avenues in the form of novel therapeutic strategies, such as gene therapy. Parallel progress in regenerative medicine indicates that TM-derived or targeted stem and progenitor cells may restore function by repopulating injured cells and reorganizing the ECM. These findings, in concert, provide a basis for mechanism-based, disease-modifying treatments [4].

In this review, we highlight recent advances (2021-2025) in TM research on mechanobiology, oxidative stress, ion channel physiology, cellular senescence, and regenerative approaches. We also incorporate seminal results from previous studies that remain relevant to contemporary understanding. Through integration of these advances, we seek to highlight the translational value of molecularly targeted treatments for glaucoma and to identify promising directions, such as gene-based therapies that can redefine the long-term treatment of this vision-debilitating disease.

## Review

Importance of trabecular meshwork in maintaining IOP

The TM, located at the iridocorneal angle, controls aqueous humor drainage via a finely tuned balance of ECM organization, cell contractility, and signaling networks. It is composed of uveal, corneoscleral, and JCT layers, with the JCT being the principal site of outflow resistance. TM cells sense mechanical stimuli from IOP fluctuations and remodel ECM accordingly, ensuring stable fluid dynamics and protecting the optic nerve head from pressure-induced damage [2,3]. Beyond its structural composition, the TM serves as an adaptive interface, responding to pulsatile aqueous humor flow and systemic hemodynamic changes. It integrates biomechanical inputs with molecular signaling to fine-tune ECM composition and cellular contractility. This dynamic interplay helps prevent acute IOP spikes and maintains a stable optic nerve perfusion environment, which is critical for retinal ganglion cell survival.

Limitations of current IOP-lowering therapies

First-line pharmacologic agents-prostaglandin analogs, β-blockers, and carbonic anhydrase inhibitors-primarily act on ciliary body aqueous humor production or uveoscleral outflow, not on the diseased TM itself. Surgical and minimally invasive glaucoma surgeries (MIGS) bypass or enhance outflow pathways but may induce inflammatory and fibrotic responses that compromise long-term efficacy. None directly reverse the cellular and molecular pathology within the TM [4]. Furthermore, long-term use of pharmacologic agents may induce compensatory changes in non-target tissues, such as alterations in conjunctival fibroblasts, potentially impacting future surgical outcomes. Similarly, while MIGS techniques have revolutionized minimally invasive IOP reduction, postoperative scarring and foreign body responses remain significant barriers to durable success, especially in younger or more inflammatory phenotypes.

Why molecular biology offers new avenues for treatment

Deciphering the TM’s molecular machinery opens the door to targeted therapies that restore physiologic outflow regulation. Mechanistic studies reveal that mechanical stress sensing, ECM turnover, and oxidative stress responses are all amenable to genetic or pharmacologic modulation. Advances in clustered regularly interspaced short palindromic repeats (CRISPR)-Cas9, RNA interference, and biologics now allow precise reprogramming of TM cell behavior [5-7]. A molecular approach allows identification of druggable targets within TM-specific pathways, such as integrin signaling, ECM crosslinking enzymes, and oxidative stress mediators. Therapeutic modulation at these points could restore TM compliance and fluid conductance without bypassing or damaging native outflow structures, potentially offering a disease-modifying effect.

Molecular architecture of the trabecular meshwork

Key Cell Types

TM endothelial-like cells are highly specialized for mechanosensing and phagocytosis. JCT cells are embedded within the ECM and regulate hydraulic conductivity. Collagens, elastin, fibronectin, and glycosaminoglycans form a dynamic scaffold. The turnover of these ECM components is not static but finely regulated by cellular feedback loops. Alterations in ECM elasticity directly influence TM cell morphology, intracellular tension, and even nuclear gene expression via mechanotransduction pathways, thereby linking structural integrity with transcriptional control of homeostasis. Matrix metalloproteinases (MMPs) and tissue inhibitors of metalloproteinases (TIMPs) coordinate ECM degradation and synthesis [8]. Crosslinking enzymes such as lysyl oxidase (LOX) increase ECM stiffness, contributing to elevated outflow resistance in glaucoma [9].

Mechanotransduction in IOP regulation

**Figure 1:**
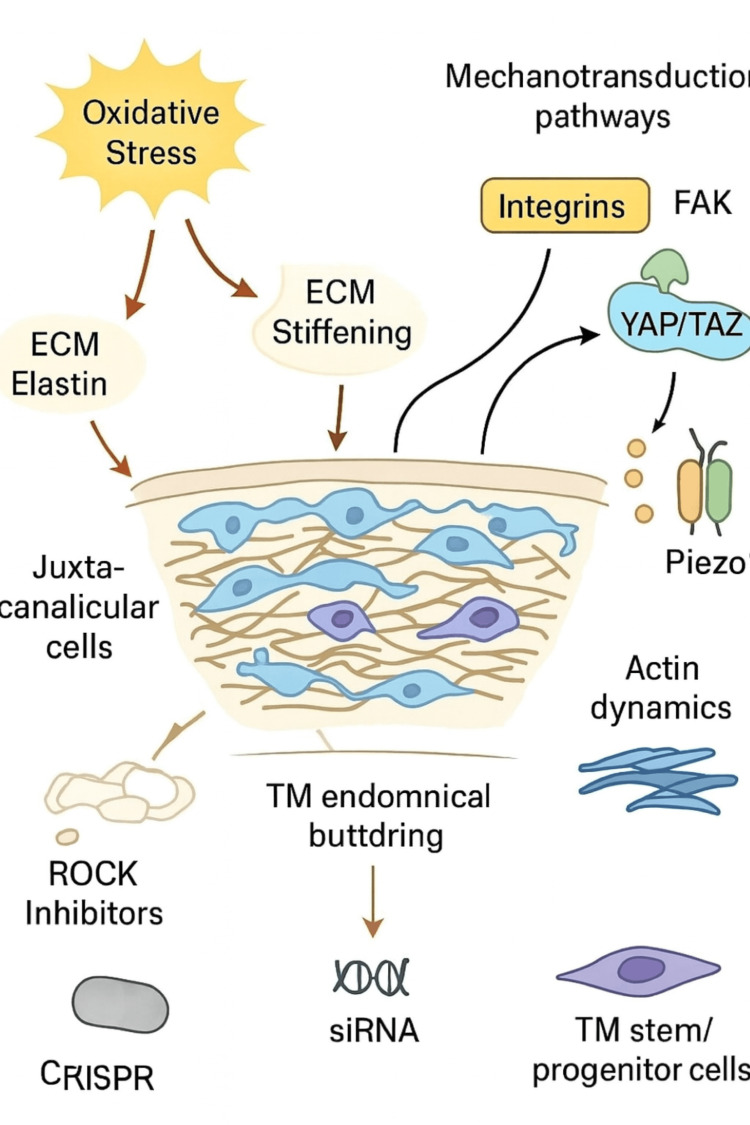
Diagram showing trabecular meshwork structure, key molecular pathways (mechanotransduction, ECM regulation, oxidative stress), and emerging therapies (ROCK inhibitors, CRISPR, siRNA, stem cells) that influence aqueous humor outflow and intraocular pressure Molecular biology of the TM in maintaining intraocular pressure. This schematic illustrates the major molecular and cellular mechanisms that regulate TM function and control aqueous humor outflow. Oxidative stress can lead to ECM stiffening, partly through ECM proteins such as elastin, which compromises TM performance. Mechanotransduction pathways - mediated by integrins, FAK, and transcriptional regulators, YAP/TAZ - influence cytoskeletal organization, while mechanosensitive ion channels such as Piezo1 fine-tune actin filament dynamics. Therapeutic strategies include pharmacological inhibition of ROCK, gene silencing using siRNA, genome editing through CRISPR, and regenerative approaches involving TM stem/progenitor cells. Together, these pathways and interventions highlight the diverse molecular targets for restoring TM health and maintaining IOP homeostasis. This figure is an original illustration created by the authors based on information synthesized from previously published studies [2,3,5,6,8,9,10,11]. CRISPR - clustered regularly interspaced short palindromic repeats; ECM - extracellular matrix; FAK - focal adhesion kinase; IOP - intraocular pressure; Piezo1 - piezo-type mechanosensitive ion channel component 1; ROCK - Rho-associated protein kinase; siRNA - small interfering ribonucleic acid; TM - trabecular meshwork; YAP/TAZ - Yes-associated protein/transcriptional coactivator with PDZ-binding motif

TM cells are constantly exposed to pressure and flow changes, and they adapt to these cues through interconnected mechanotransduction pathways. Integrins, together with focal adhesion kinase (FAK), act as anchors and sensors that detect ECM stiffness and transmit these signals to the actin cytoskeleton, initiating downstream cascades such as Rho/ROCK and MAPK [10]. These mechanical inputs converge on YAP/TAZ, transcriptional regulators that shuttle between the cytoplasm and nucleus depending on cytoskeletal tension. Once activated, they govern gene networks linked to cell growth, ECM remodeling, and cytoskeletal organization. Mechanosensitive ion channels, particularly TRPV4 and Piezo1, provide another level of control. By detecting stretch and pressure, they facilitate calcium influx, which drives cytoskeletal contractility and coordinates with mitochondrial activity to meet the energy demands of sustained mechanical load [2,3,11]. These ion channels, in turn, interact with integrin- and cytoskeleton-driven signaling, indirectly influencing YAP/TAZ activity. Together, these systems form an integrated network: integrins and FAK connect ECM stiffness to cytoskeletal remodeling; calcium influx via TRPV4 and Piezo1 fine-tunes cytoskeletal and mitochondrial responses; and YAP/TAZ convert these signals into long-term transcriptional programs. Cytoskeletal dynamics, particularly actin stress fiber assembly, serve as the structural endpoint of these pathways, altering cell shape and aqueous humor outflow resistance. When this balance is disturbed, as in glaucoma, TM cells undergo maladaptive stiffening, aberrant ECM deposition, and impaired aqueous outflow (Table 1, Figure 1). Although integrins, FAK, YAP/TAZ, TRPV4, and Piezo1 are well recognized as core mechanosensors, comparative quantitative data on their relative contributions remain sparse. Future studies with high-resolution, quantitative approaches will be essential to clarify their individual and cooperative roles in glaucoma pathogenesis.

**Table 1:** Key molecular pathways in trabecular meshwork mechanotransduction Piezo1 - piezo-type mechanosensitive ion channel component 1; YAP/TAZ - Yes-associated protein/transcriptional coactivator with PDZ-binding motif

Pathway/Component	Function in TM	Evidence From Studies	Therapeutic Implications	Reference(s)
Integrins and FAK	Link ECM stiffness to cytoskeletal remodeling	Altered integrin signaling in glaucomatous TM	Targeting integrin-FAK to modulate stiffness	[10]
YAP/TAZ	Transcriptional control in response to strain	Dysregulated in stiffened ECM	YAP inhibitors or activators	[2,3,11]
TRPV4 channel	Calcium influx under mechanical load	TRPV4-eNOS pathway impaired in glaucoma	TRPV4 agonists/antagonists	[2,3,10]
Piezo1 channel	Senses stretch, regulates aqueous outflow	Promotes outflow via calcium-mediated cytoskeletal change	Potential channel modulators	[3,11]

Pathophysiology of disease

Oxidative Stress, Senescence, and ECM Stiffening

The interplay of oxidative stress, cellular senescence, and ECM remodeling forms a central axis in TM dysfunction and glaucoma pathogenesis. Oxidative stress leads to the accumulation of reactive oxygen species (ROS), which damage DNA, proteins, and lipids in TM cells, ultimately driving apoptosis and loss of function [6]. Prolonged oxidative stress also impairs mitochondrial ATP production, leaving cells metabolically compromised and prone to senescence.

In the senescent state, TM cells adopt a senescence-associated secretory phenotype (SASP), characterized by the release of pro-inflammatory cytokines, growth factors, and proteases. This secretory profile perpetuates ECM fibrosis and inflammation, further stiffening the outflow pathway [12-15]. The resulting ECM stiffening is reinforced by increased LOX activity and reduced MMP activity, which together decrease ECM turnover and elasticity [15]. As the TM becomes rigid, its ability to sense and respond to mechanical cues diminishes, creating a feed-forward loop of maladaptation that progressively elevates IOP.

While this framework provides a strong molecular explanation for glaucoma pathogenesis, the section has now been strengthened by incorporating recent biomarker studies. These include investigations of oxidative stress markers (such as 8-OHdG and malondialdehyde) and senescence-associated proteins, which are increasingly being evaluated in aqueous humor and TM tissue. Such biomarkers highlight the translational potential of linking molecular mechanisms to early detection and prognosis in glaucoma (Table 2) [16]. 

**Table 2:** Pathophysiologic changes in TM in disease states ECM - extracellular matrix; LOX - lysyl oxidase; MMP - matrix metalloproteinase; SASP - senescence-associated secretory phenotype; TM - trabecular meshwork

Mechanism	Key Changes	Evidence	Resulting Effect	Reference(s)
Oxidative stress	ROS accumulation, mitochondrial damage	In vitro oxidative models	TM apoptosis, senescence	[12,14]
Senescence (SASP)	Inflammatory cytokine release	Senescent TM shear stress response	ECM fibrosis, stiffness	[13,14]
ECM stiffening	↑ LOX, ↓ MMP activity	Human glaucoma tissue	Reduced mechanosensing, increased resistance	[9,15]

Molecular targets for therapy

ROCK inhibitors relax the TM cytoskeleton, thereby lowering outflow resistance. These agents disrupt actin stress fiber formation, enhancing TM outflow capacity. Gene therapies, whether through CRISPR-mediated mutation correction or RNA-based suppression of pro-fibrotic genes, are showing preclinical promise. Cell-based approaches leverage the regenerative potential of TM stem/progenitor cells, which can integrate into host tissue, secrete healthy ECM, and restore mechanosensitivity. Combining these modalities with targeted drug delivery systems could provide long-lasting restoration of TM physiology [5].

Recent advances in gene and cell therapy have created opportunities to directly target TM dysfunction in glaucoma. Gene therapy approaches such as CRISPR-Cas9 have been used experimentally to correct MYOC mutations that cause misfolded protein accumulation and TM cell death [6]. Similarly, siRNA-based therapies can silence TGF-β2-induced fibrotic gene expression, thereby reducing excessive ECM deposition and stiffness [8]. A central challenge for these strategies is the mode of delivery. Viral vectors, particularly adeno-associated viruses (AAVs), are currently the most efficient means of transferring genes to TM cells, but their use raises concerns regarding immunogenicity, off-target effects, and long-term safety. To address these issues, non-viral platforms such as lipid nanoparticles and polymer-based carriers are being developed, although further optimization is needed to achieve targeted, sustained expression within the outflow pathway.

Cell-based therapies are also emerging as a promising approach. Studies using TM stem/progenitor cells and induced pluripotent stem cells (iPSCs) suggest that transplanted cells can integrate into the TM, restore phagocytic activity, and enhance aqueous outflow. However, significant safety concerns remain, including the risk of aberrant differentiation, immune rejection, and uncontrolled proliferation. Ensuring both the genomic stability and functional integration of transplanted cells is critical before these strategies can move into clinical practice. Overall, while gene and cell therapies hold great promise for restoring TM function rather than simply lowering IOP, their successful translation will depend on continued refinement of delivery platforms and rigorous evaluation of long-term safety (Table 3).

**Table 3:** Emerging molecular and cellular therapies for TM restoration CRISPR - clustered regularly interspaced short palindromic repeats; ECM - extracellular matrix; IOP - intraocular pressure; siRNA - small interfering ribonucleic acid; TGF-β2 - transforming growth factor beta 2; TM - trabecular meshwork

Therapy Type	Target	Mode of Action	Preclinical/Clinical Evidence	Reference(s)
ROCK inhibitors	Cytoskeleton	Relax actin stress fibers	Clinical use in IOP lowering	[16]
CRISPR-Cas9 gene editing	MYOC mutation	Corrects pathogenic variants	Preclinical proof-of-concept	[6]
siRNA	TGF-β2	Silences fibrotic gene expression	In vitro human TM	[8]
TM stem/progenitor cells	Damaged TM	Repopulation, ECM secretion	Transcriptomic profiling, in vivo	[5,6]

Impact of MIGS on trabecular meshwork biology

Preliminary clinical reports of perioperative adjuncts, such as localized anti-fibrotic regimens and short-course ROCK-inhibitor therapy, suggest potential early improvements in outflow and medication burden following MIGS; however, these studies are limited by small cohorts, variable adjunct protocols, and short follow-up periods. Gene-modulatory strategies and biodegradable, drug-eluting stents remain investigational, with first-in-human or feasibility data but no definitive long-term outcomes. Accordingly, while perioperative molecular modulation is biologically compelling, confirmation of durable efficacy and safety will require adequately powered randomized trials with standardized success criteria (Table 4).

**Table 4:** Potential perioperative molecular adjuncts for MIGS ECM - extracellular matrix; MIGS - minimally invasive glaucoma surgery; ROCK - Rho-associated protein kinase; TGF-β - transforming growth factor beta; TM - trabecular meshwork

Therapy/Adjunct	Mechanism of Action	Targeted Issue	Level of Evidence	Preclinical/Clinical Evidence	Reference(s)
Anti-TGF-β agents	Reduce fibrosis	Post-surgical ECM overproduction	Early clinical and animal (Levels II-III)	Demonstrated efficacy in animal models; early human studies show reduced scarring	[4]
ROCK inhibitors (local delivery)	Relax trabecular meshwork (TM)	Enhance outflow post-device (MIGS)	Preclinical (Level IV)	Local application in preclinical models improves outflow	[16]
Drug-eluting stents	Controlled release of antifibrotic or anti-inflammatory molecules	Prevent inflammation and scarring after surgery	Preclinical (Level IV)	Evaluated in laboratory and animal models	[4]

Future directions

Emerging tools such as single-cell RNA sequencing and proteomics are beginning to unravel the complexity of TM biology by identifying distinct cell subtypes and molecular signatures in health versus disease [17]. This systems-level mapping has the potential to enable more precise classification of glaucoma subtypes, moving beyond IOP as the sole diagnostic benchmark. At the same time, integrating such insights into clinical workflows will depend on the development of rapid, cost-effective molecular diagnostic technologies that are not yet widely available in routine ophthalmic practice. Personalized molecular profiling, in which therapeutic strategies are tailored to patient-specific genetic and proteomic signatures, represents an attractive but still aspirational goal that will require validation through large-scale longitudinal studies. Likewise, combination strategies - pairing molecular approaches such as siRNA or CRISPR with MIGS - hold promise for synergistic TM restoration, but their safety, durability, and cost-effectiveness remain to be fully established. Taken together, these directions highlight exciting opportunities, yet underscore the need for careful preclinical validation, long-term clinical trials, and pragmatic consideration of accessibility before they can be widely adopted in practice.

## Conclusions

A paradigm shift in glaucoma management is emerging from pressure-lowering to pressure-normalizing via molecular restoration of TM function. By targeting the mechanotransduction pathways, ECM homeostasis, and cellular viability of the TM, future therapies may halt or reverse disease progression, preserving vision and quality of life. Future glaucoma management may adopt a hybrid model, combining early detection of molecular derangements with targeted biologic or genetic interventions, ensuring preservation of vision well before functional loss manifests in standard perimetry or OCT.
